# Impact of self-identity on social anxiety among college students: a moderated mediation model

**DOI:** 10.3389/fpsyg.2025.1622431

**Published:** 2025-07-30

**Authors:** Shuqi Guo, Jiazhong Yang, Shicong Zhang, Dongzhuo Xue, Mingxu Liu

**Affiliations:** Institute of Flight Technology, Civil Aviation Flight University of China, Guanghan, China

**Keywords:** social anxiety, self-identity, negative evaluations, social support, college students

## Abstract

**Background:**

Social anxiety is a prevalent and impairing condition among college students, often interfering with academic performance, emotional wellbeing, and social functioning.

**Objective:**

This study aimed to test a moderated mediation model in which self-identity predicts social anxiety, with fear of negative evaluation (FNE) serving as a mediator and perceived social support acting as a moderator in the latter part of the pathway.

**Methods:**

A total of 328 college students (Mage = 19.48, SD = 1.02; 43.29% male) completed standardized self-report measures of self-identity, FNE, social anxiety, and perceived social support. Structural equation modeling (SEM) was used to examine the proposed model.

**Results:**

Self-identity significantly negatively predicted social anxiety (β = −0.55, *p* < 0.001). FNE partially mediated this relationship, accounting for about half of the total effect. Furthermore, perceived social support weakened the association between FNE and social anxiety under high-support conditions (moderated effect: β = −0.14, *p* < 0.01).

**Conclusion:**

These findings suggest the potential value of integrative intervention strategies that promote self-identity, reduce fear of negative evaluation, and enhance perceived social support to mitigate social anxiety in college populations.

## 1 Introduction

### 1.1 Social anxiety disorder among college students

Social anxiety disorder (SAD), as defined in the Diagnostic and Statistical Manual of Mental Disorders (DSM-5), refers to an intense and persistent fear of negative evaluation in social or performance situations (Rapee and Heimberg, 1997). With a lifetime prevalence of approximately 17% and increasing incidence during adolescence and early adulthood, SAD is one of the most common psychiatric disorders among college students (Salari et al., 2024). The disorder is associated with significant impairments in academic functioning, psychological wellbeing, and social engagement (Buckner et al., 2017; Lizarte Simón et al., 2024). Moreover, the age-related gradient from 4.7% in childhood to 17% in adolescence underscores young adulthood as a critical opportunity for intervention (Salari et al., 2024). However, recent studies underscore the importance of individual differences in psychological traits and contextual moderators in shaping vulnerability to SAD (Rapee and Heimberg, 1997). In particular, self-identity, fear of negative evaluation (FNE), and perceived social support have emerged as key psychological constructs influencing the development and maintenance of social anxiety.

### 1.2 Self-identity and social anxiety

Self-identity refers to an individual’s integrated acceptance of personal values and social roles (Schreurs, 2001). Research has shown that stable self-identity forms the psychological foundation for coping with social challenges (Schreurs, 2001). Rooted in Erikson’s psychosocial development theory, self-identity formation during adolescence and early adulthood is critical for fostering self-esteem, social belonging, and mental health. Drawing on Marcia’s identity status model, self-identity can be understood as the result of both exploration and commitment, which together determine the individual’s coherence and direction in life. While deficits in self-identity have been associated with social anxiety, the nature of this relationship is complex. Impaired self-evaluation may contribute to increased sensitivity in socially evaluative contexts (Rapee and Heimberg, 1997), yet it is also possible that prolonged social withdrawal limits identity development through reduced social learning opportunities. Therefore, the association between self-identity and social anxiety may be bidirectional and mutually reinforcing. Recent research also indicates that students with high levels of social anxiety, even without a diagnosis of autism, tend to show deficits in adaptive conceptual and social functioning, suggesting that weak self-identity may interact with broader impairments in daily functioning (Zukerman et al., 2025). Individuals with low self-identity are more likely to misinterpret neutral social cues as threatening, which causes avoidance behavior. Longitudinal data have indicated that self-identity positively predicts self-esteem stability through identity importance (β = 0.32, *p* < 0.01) and identity commitment (Chen, 2019). These regression coefficients suggest that identity strength supports a stable self-concept over time, thereby reducing vulnerability to internalizing symptoms. A meta-analysis further revealed that in individuals with low self-identity, self-esteem deficits increased the risk of social anxiety by 72% (OR = 1.72), which was significantly higher than the overall effect size (β = −0.10) (Sowislo and Orth, 2013). These findings suggest that self-identity may buffer individuals from social threats by maintaining stable self-esteem.

### 1.3 The mediating role of fear of negative evaluation

FNE is defined as a persistent concern regarding potential negative judgments during social interactions (Watson and Friend, 1969). Its core features are explained by a dual-process model: FNE increases vigilance toward threat cues (β = 0.41, *p* < 0.01) while simultaneously suppressing the processing of positive social signals, thereby reinforcing a maladaptive vigilance–avoidance cycle (Weeks and Howell, 2012). Empirical evidence indicates that each one-standard-deviation increase in FNE is associated with a 37% greater risk of social avoidance among college students (OR = 1.37, 95% CI [1.12, 1.68]), with stronger effects in social anxiety subgroups (ΔOR = 0.28, *p* = 0.003) (Villarosa-Hurlocker et al., 2018). Clinical studies further confirmed that FNE was positively associated with negative interpretation bias toward ambiguous cues among individuals with social anxiety disorder (*r* = 0.62, *p* < 0.001), and such subjective threat perception predicts anxiety more strongly than objective context (ΔR^2^ = 0.19) (Weeks et al., 2005). From a cognitive-behavioral perspective, FNE maintains social anxiety through two major mechanisms. First, it increases self-focused attention by activating catastrophic beliefs regarding interpersonal rejection, which leads to attentional fixation on perceived personal flaws (Voncken et al., 2010). Second, it distorts cognitive schemas. Individuals with chronically high FNE often develop core beliefs regarding an “unacceptable self,” which fosters overreliance on safety behaviors and ultimately reinforces anxiety (Borkovec and Newman, 1998). This perspective aligns with chained mediation models increasingly used in psychological research. For example, previous study demonstrated that physical exercise reduces college students’ social anxiety through a chain mediating pathway involving mindfulness and mental toughness, providing empirical support for the cognitive–emotional route of anxiety mitigation (Jiang et al., 2025).

### 1.4 The moderating role of perceived social support

Perceived social support refers to an individual’s subjective evaluation of the support received from family, peers, and other social relationships (Sarason et al., 1991). Perceived social support can disrupt these maladaptive mechanisms through two primary approaches: (1) Primary appraisal reappraisal: High levels of social support encourage individuals to reinterpret ambiguous social cues as neutral or constructive (Cohen and Wills, 1985). For instance, it can enhance self-efficacy (β = 0.32, *p* < 0.01), thereby reducing excessive vigilance toward negative evaluation (Wood et al., 2008). (2) Secondary coping activation: Supportive networks provide emotional resources and problem-solving strategies, thereby interrupting the FNE → self-criticism → social avoidance cascade (β = −0.33, *p* < 0.01) (Wong et al., 2014). Importantly, self-identity played a synergistic role in this process. Individuals with higher self-identity use support resources more effectively through stable self-schemas (moderation effect: β = 0.27, *p* = 0.008), enhancing the interruption of the FNE-to-anxiety pathway. These findings are consistent with the stress-buffering model, which posits that social support weakens the predictive power of FNE on social anxiety by modulating threat appraisal and the allocation of coping resources (ΔR^2^ = 0.18) (Cohen and Wills, 1985).

Its anxiety-buffering effects can be explained from two theoretical perspectives: Social cognitive theory and the stress buffering model. Social cognitive theory emphasizes the role of observational learning and appraisal processes in guiding behavior. Individuals with high perceived support tend to reinterpret socially ambiguous situations—such as public speaking—as opportunities for authentic self-expression rather than performance threats (Wood et al., 2008). Compared to individuals with low perceived support, those with higher support levels showed significantly lower rates of social avoidance (χ^2^ = 6.32, *p* = 0.012). Meanwhile, the stress-buffering model (Cohen and Wills, 1985) frames perceived support as a context-sensitive moderator that operates through appraisal and coping routes. Specifically: (1) Primary appraisal regulation reduces catastrophic interpretation of neutral cues, such as ambiguous facial expressions. (2) Secondary coping enhancement: This increases adaptive behaviors such as clarification, problem-solving, and emotional regulation (Lakey and Orehek, 2011; Cohen and Wills, 1985). Recent extensions to the stress-buffering model emphasize the importance of individual differences in receptivity to support (Uchino, 2009), providing additional theoretical justification for the moderating role of self-identity. People with high identity coherence are more likely to perceive support as helpful and congruent with their self-view, enhancing its protective effects.

### 1.5 Theoretical integration and model hypothesis

#### 1.5.1 Prior findings

Previous research on social anxiety has primarily focused on internal psychological mechanisms, particularly the formation and stabilization of self-identity. Leary (1983) suggested that deficits in self-schema can heighten sensitivity to social threat and promote anxiety. Interventions rooted in this “inside-out” approach often emphasize building self-coherence to reduce vulnerability. In parallel, the stress-buffering model (Cohen and Wills, 1985) has highlighted the protective role of social support in regulating stress responses. This model emphasizes that external support systems can influence threat appraisal, enhance coping capacity, and reduce maladaptive emotional responses.

#### 1.5.2 Theoretical rationale

Integrating these perspectives, this study proposes a dual-pathway model that combines internal cognitive structures with external regulatory factors. Specifically, self-identity is positioned as a distal predictor of social anxiety, operating through fear of negative evaluation (FNE) as a cognitive mediator. At the same time, perceived social support is introduced as a moderator that buffers the impact of FNE on social anxiety. This design is grounded in the cognitive-behavioral framework, which explains how dysfunctional self-evaluation and threat anticipation maintain social anxiety (Hofmann, 2007). It has been hypothesized that high levels of perceived social support attenuate the predictive effect of FNE on social anxiety, potentially by facilitating the positive reinterpretation of ambiguous social cues (Moscovitch, 2009).

#### 1.5.3 Unique contribution

This study contributes to the literature by: bridging internal psychological mechanisms (self-identity, FNE) and external contextual factors (social support); Introducing a moderated mediation model that reflects complex, real-world regulatory processes; providing a theoretical foundation for integrated interventions that coordinate both personal and interpersonal resilience mechanisms.

#### 1.5.4 Model hypotheses

**FIGURE 1:**
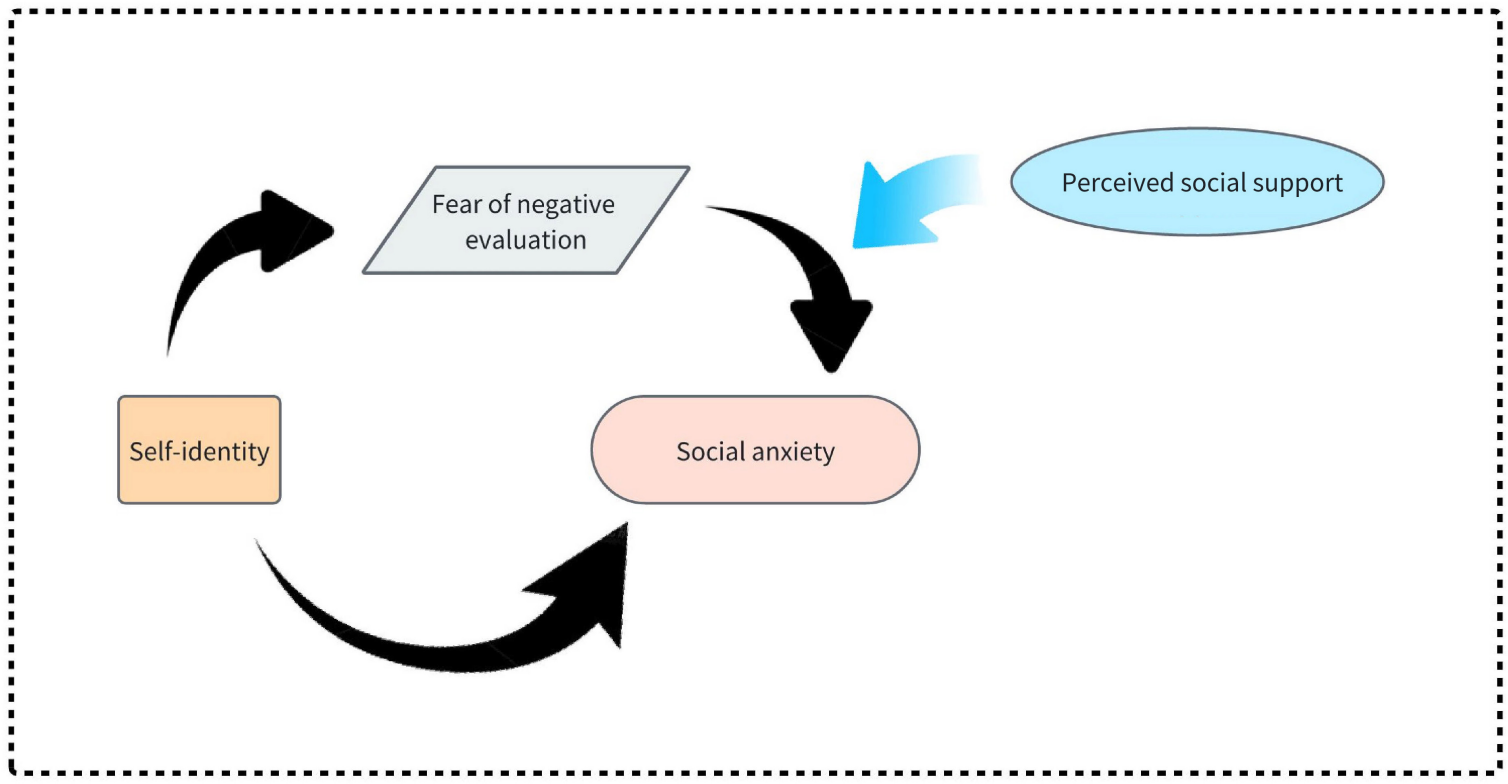
Hypothetical model of moderated mediating effects.

The hypothesized model is illustrated in Figure 1. Specifically, we hypothesize:

H1: Self-identity negatively predicts social anxiety;
H2: FNE mediates the relationship between self-identity and social anxiety;
H3: Perceived social support moderates the association between FNE and social anxiety, such that the relationship is weaker under high-support conditions.

## 2 Materials and methods

### 2.1 Participants

*A priori* power analysis was conducted using G*Power version 3.1.9.7 (Faul et al., 2007) to determine the adequacy of the sample size. The results indicated that a sample size of 119 participants was required to detect a medium effect size (*d* = 0.30) with 99% statistical power (1-β = 0.99) at a significance level of α = 0.05. A cluster sampling method was used to minimize the environmental measurement errors. A total of 350 students were recruited from Liaocheng University, Shandong Province, China, to complete a paper-based questionnaire. After excluding invalid responses, 328 valid responses were collected, resulting in a response rate of 93.71%. The participants had a mean age of 19.48 years (SD = 1.02), ranging from 17 to 23 years, and 56.71% were female.

### 2.2 Materials

#### 2.2.1 Self-identity scale

The scale consists of 19 items rated on a 4-point Likert scale ranging from one (not at all applicable) to four (very applicable). Items 1, 2, 4, 8, 9, 12, 13, 14, 15, 16, 17, and 18 were reverse-scored. Subsequently, the item responses were summed to calculate the total score, with higher scores indicating a greater level of self-identity development. Cronbach’s alpha for the scale was 0.74.

#### 2.2.2 Short fear of negative evaluation scale

The scale, revised by Leary (1983), consists of 12 items rated on a 5-point Likert scale ranging from one (not at all true of me) to five (very true of me). Items 2, 4, 7, and 10 were reverse-scored. Subsequently, the item responses were summed to compute a total score, with higher scores indicating a greater FNE. Cronbach’s alpha coefficient for the scale was 0.85.

#### 2.2.3 Perceived social support scale

The scale consists of 12 items rated on a 7-point Likert scale ranging from one (strongly disagree) to seven (strongly agree). It comprises three subscales: family, friends, and support from significant others. Total scores were computed, with higher scores indicating higher levels of perceived social support across each aspect. Cronbach’s alpha coefficients for the family, friend, and other significant subscales were 0.83, 0.88, and 0.79, respectively.

#### 2.2.4 Interaction anxiousness scale

This scale, developed by Leary (1983), consists of 15 items rated on a 5-point Likert scale ranging from one (not at all characteristic of me) to five (very characteristic of me). Items 3, 6, 10, and 15 were reverse-scored. Subsequently, the item responses were summed to calculate the total score, with higher scores indicating greater social anxiety. Cronbach’s alpha for the scale was 0.85.

### 2.3 Data analysis

Data analysis was performed using SPSS version 24.0. First, Harman’s single-factor test was used to assess the common method bias (CMB). Descriptive statistics and Pearson’s correlation analyses were conducted to explore the relationships between the study variables. Finally, confirmatory factor analysis (CFA) and structural equation modeling (SEM) were conducted using Mplus to examine the relationships between the latent variables. An item parceling strategy was used to improve the model estimation.

## 3 Results

### 3.1 Common method deviation test

The potential for CMB was considered because all data were collected through self-report measures (Podsakoff et al., 2003). Harman’s single-factor test was used to assess the presence of CMB. The unrotated factor analysis extracted four factors with eigenvalues greater than one. The first factor accounted for only 25.50% of the total variance, which was below the critical threshold of 40%. Therefore, the CMB was not a serious concern.

### 3.2 Descriptive and correlation analysis

Partial correlation analyses were conducted to examine the relationships between the main variables while controlling for sex, age, and grade level. Table 1 presents the results of the study. Specifically, self-identity was significantly positively correlated with perceived social support and negatively correlated with social anxiety and FNE. Social anxiety was significantly and positively correlated with FNE and negatively correlated with perceived social support.

**TABLE 1:** Descriptive statistics and partial correlation analysis of each variable.

Variable	M	SD	1	2	3	4
Self-identity	58.85	6.20	–			
Social anxiety	45.01	9.12	−0.45***	–		
Fear of negative evaluations	40.53	7.73	−0.29***	0.54***	–	
perceived social support	63.35	11.25	0.48***	−0.27***	0.04	–

***p < 0.001. Relationship between self-identity and social anxiety: a moderated mediation model test.

Based on the item parceling strategy, a balanced approach of the factor method was used to create parcels for each construct, forming observed indicators for the latent variables (Little et al., 2002). As shown in Table 2, three parcels were developed for each latent variable: self-identity, social anxiety, FNE, and perceived social support. The correlations between each parcel and its corresponding latent construct ranged from 0.61 to 0.92, which were statistically significant and high. These results indicate that the parcels adequately represented their respective latent constructs.

**TABLE 2:** Matrix of correlation coefficients between measured indices and studied variables.

Study variables	Item 1	Item 2	Item 3
Self-identity	0.76	0.78	0.61
Social anxiety	0.81	0.80	0.77
Fear of negative evaluations	0.79	0.91	0.75
perceived social support	0.87	0.92	0.89

**FIGURE 2:**
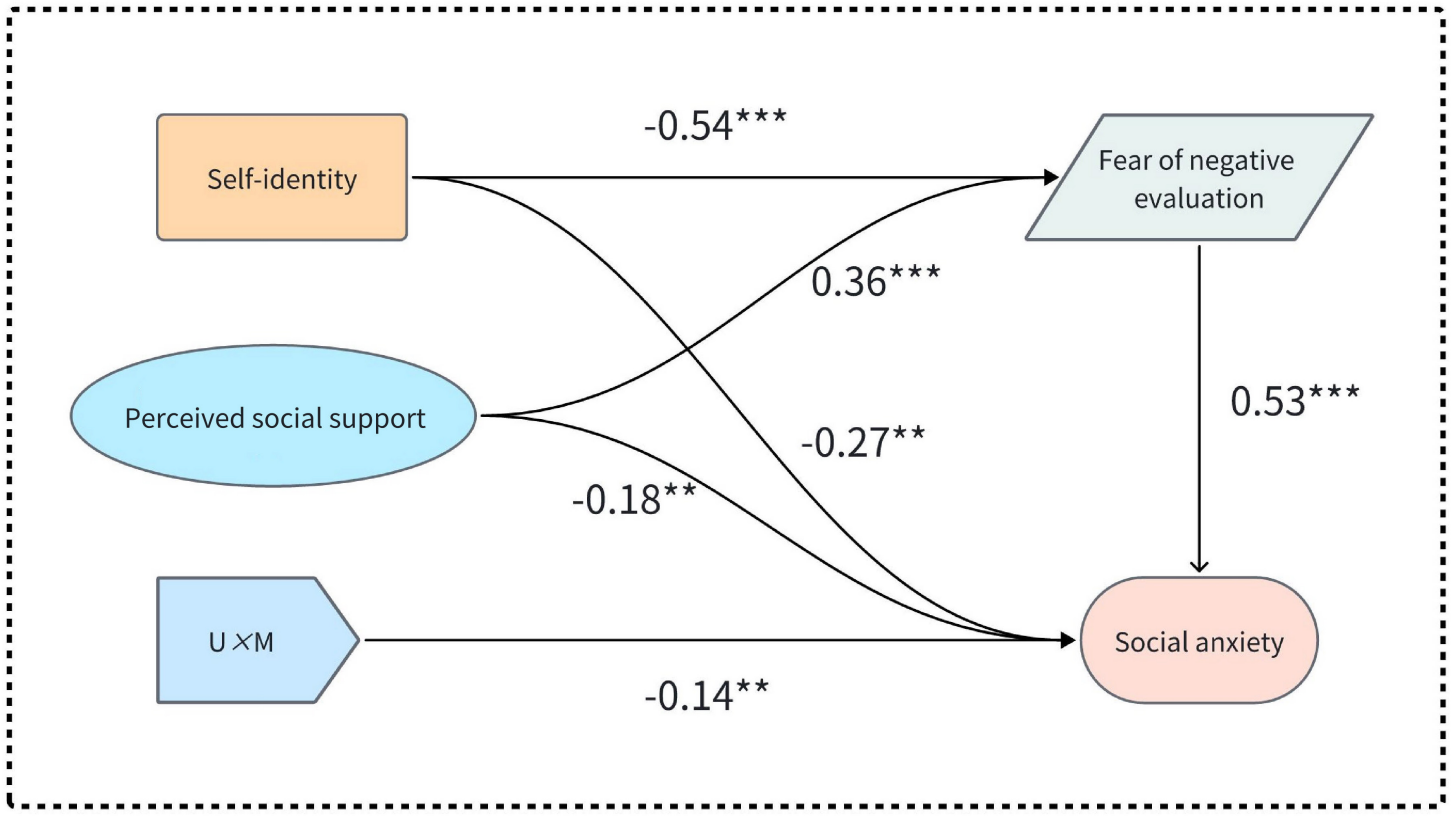
Mediation model diagram with moderation. **p < 0.01, ***p < 0.001.

A CFA was conducted using Mplus, including only self-identity and social anxiety in the structural equation model. Self-identity significantly negatively predicted social anxiety among college students (β = −0.55, *t* = −10.43, *p* < 0.001). The model showed a good fit: χ^2^/df = 1.57, TLI = 0.99, CFI = 0.99, RMSEA = 0.04, SRMR = 0.03. These results confirmed the negative predictive effect of self-identity on social anxiety. Subsequently, the matched-product method was applied to compute an interaction term by multiplying the standardized scores for FNE and perceived social support (Marsh et al., 2004). Subsequently, the structural equation model was tested. As shown in Figure 2, the model demonstrated a good fit. Fit indices were as follows: χ^2^/df = 1.63, TLI = 0.97, CFI = 0.98, RMSEA = 0.04, and SRMR = 0.04. Self-identity significantly negatively predicted FNE (β = −0.54, *t* = −7.15, *p* < 0.001). FNE significantly positively predicted social anxiety (β = 0.53, *t* = 9.34, *p* < 0.001). These results indicate that the FNE mediates the relationship between self-identity and social anxiety. The indirect effects accounted for 51.45% of the total effects. Additionally, self-identity remained a significant predictor of social anxiety (β = −0.27, *t* = −3.361, *p* < 0.01), indicating a partial mediating effect of FNE in the relationship between self-identity and social anxiety. Furthermore, the interaction term between FNE and perceived social support significantly predicted social anxiety (β = −0.14, *t* = −2.73, *p* < 0.01). This finding suggests that perceived social support moderates the relationship between FNE and social anxiety.

**FIGURE 3:**
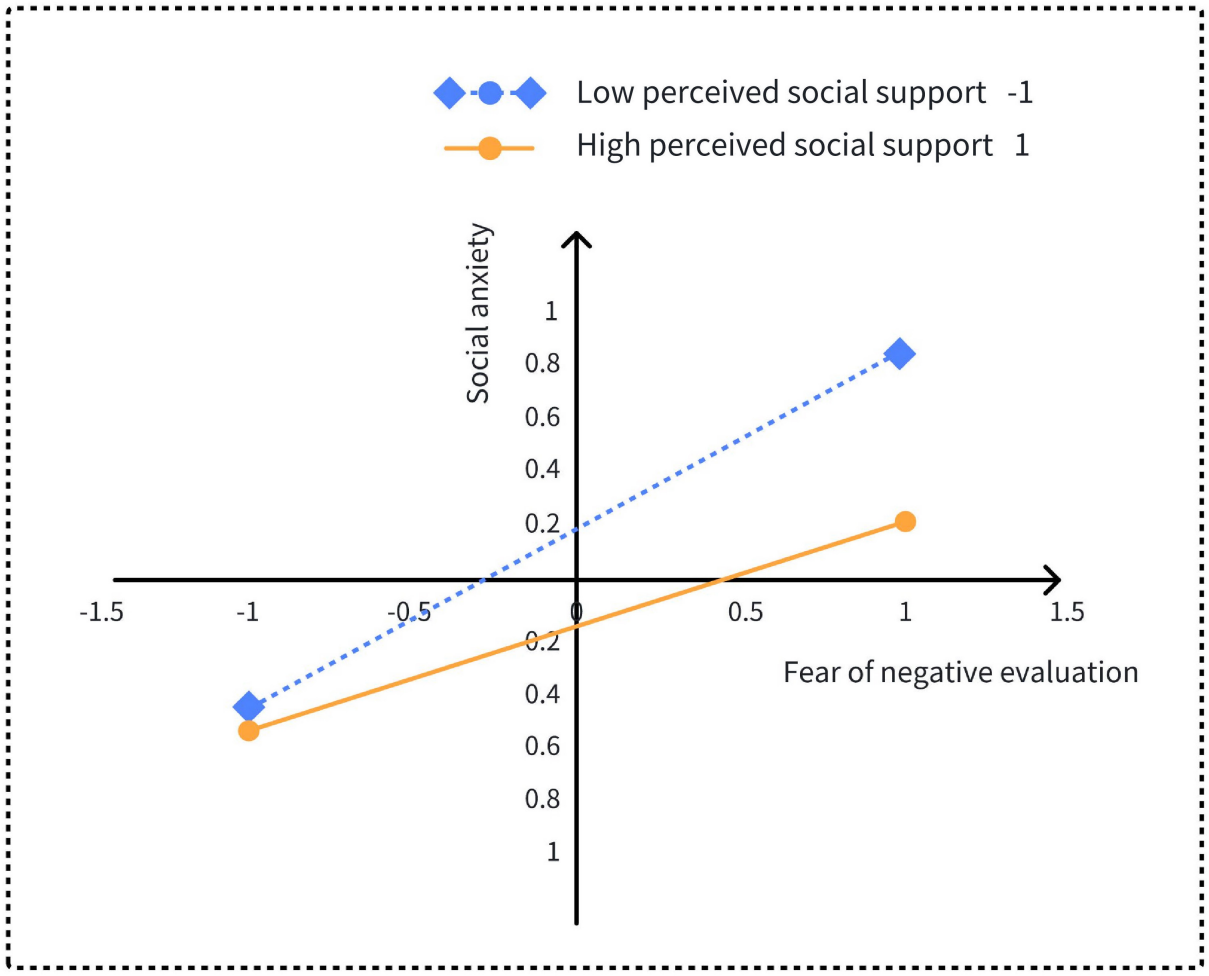
Understanding the moderation of social support on the relationship between self-identity and social anxiety.

The Z-scores of perceived social support were set at ± 1 to generate an interaction plot and a conditional indirect effects table. As shown in Figure 3 and Table 3, these results provide a comprehensive understanding of how perceived social support moderates the relationship between the FNE and social anxiety. Simple slope analyses revealed that for college students with low perceived social support (*Z* = −1), increases in FNE were positively associated with increases in social anxiety (β = 0.68, *t* = 6.46, *p* < 0.001). Specifically, a one standard deviation increase in FNE was associated with a 0.68 standard deviation increase in social anxiety. For students with high levels of perceived social support (*Z* = 1), the relationship remained significant but weaker (β = 0.39, *t* = 4.86, *p* < 0.001). In this case, a one-standard-deviation increase in FNE corresponded to only a 0.39 standard deviation increase in social anxiety.

**TABLE 3:** Mediating effect at different levels of perceived social support.

	Perceived social support	Indirect effect value	Boot standard error	Boot CI lower limit	Boot CI upper limit	Indirect/total effect
The second half of the path of the negative evaluation fear mediating effect	−1.00 (M-1SD)	−0.38	0.07	−0.58	−0.25	69.09%
	0 (M-1SD)	−0.30	0.05	−0.45	−0.20	54.55%
	1.00 (M-1SD)	−0.22	0.06	−0.36	−0.11	40%

## 4 Discussion

By integrating perspectives from social cognitive theory and the stress-buffering model, this study systematically revealed the approach through which self-identity influences social anxiety among college students as well as the boundary conditions of this effect. The findings confirmed the mediating role of FNE and further demonstrated the moderating effect of perceived social support on the cognitive–affective pathway in the context of social anxiety. These results provide a novel theoretical framework for understanding individual differences in anxiety disorders.

From the perspective of self-concept development, the direct effect of self-identity on social anxiety (β = −0.55) can be compared to the effect size typically observed in cognitive behavioral therapy within clinical samples (Weeks et al., 2008). This suggests that identity stability may function as a natural psychological buffer, enhancing metacognitive monitoring in socially evaluative contexts. Individuals with strong identity integration are more likely to interpret ambiguous social cues as neutral during primary appraisal (Mogg and Bradley, 2002), and sustain core self-worth through identity commitment in secondary appraisal (Talaifar and Swann, 2017). These mechanisms help maintain adaptive coping strategies even when facing social threat.

The results revealed a significant mediating effect of FNE on the relationship between self-identity deficiency and social anxiety, with an indirect effect accounting for 51.45% of the total effect (95% CI [38.72, 63.18]). As proposed by the dual-process model (Weeks and Howell, 2012), FNE amplifies attention toward threat cues while suppressing positive feedback processing. Individuals with low self-identity tend to internalize social rejection, fostering a hypervigilant monitoring style and negative self-schemas. Neuroimaging evidence supports this interpretation, revealing that right amygdala activation increased by approximately 18.7% in response to social evaluation, accompanied by reduced prefrontal regulatory activity (Brühl et al., 2014). These neural patterns correspond to observable social avoidance, reinforcing the anxiety cycle.

Crucially, perceived social support moderated the link between FNE and social anxiety, reducing the predictive strength of FNE by 49.1% under high support conditions. This suggests that external support may buffer anxiety by reappraising threat and enhancing coping capacity. Biologically, support has been shown to stimulate dopaminergic activation in the ventral striatum (Inagaki and Ross, 2018) and inhibit amygdala–insula hyperactivation, reducing neural threat salience by approximately 29% (Coan et al., 2006). Behavioral data reinforce this, with 73.2% of participants in the high-support group actively clarifying misunderstandings during stress tasks, compared to 34.5% in the low-support group (Bolger and Amarel, 2007). These results align with the stress-buffering hypothesis (Cohen and Wills, 1985) and point to support systems as moderators of emotional resilience.

These findings have important implications for clinical interventions. First, narrative- or values-based identity interventions could strengthen self-concept integration and reduce baseline risk (Heimberg et al., 2014). Second, targeting FNE through metacognitive restructuring may address the core cognitive vulnerability in social anxiety. Finally, structured peer support programs can enhance resilience by mitigating real-time stress responses. A multidimensional strategy addressing identity, cognition, and environment may be especially effective (Hofmann and Smits, 2008).

## 5 Limitations and future research

Directionality of causality and third-variable confounding: The cross-sectional design limits the ability to rule out reverse causality and influence of unmeasured confounding variables. For example, longitudinal research suggests that low self-identity and social anxiety may form a mutually reinforcing loop; self-identity deficits intensify social avoidance behaviors, whereas prolonged avoidance restricts opportunities to reconstruct identity through social interactions (Cole and Maxwell, 2003). Moreover, unmeasured variables such as neuroticism or early attachment trauma may simultaneously predict self-identity vulnerability and increased FNE (Spence and Rapee, 2016). Moreover, potential confounding variables such as neuroticism or early attachment trauma were not assessed in this study, though both may influence identity development and social anxiety. Future studies should incorporate personality assessments or developmental history tools to control for such effects. Future research should employ longitudinal moderation analyses to examine whether the buffering effect of perceived social support on the relationship between fear of negative evaluation (FNE) and social anxiety is stable over time Future studies could use cross-lagged panel designs with intervals of six to twelve Months to clarify the temporal sequence of the variables. Alternatively, microintervention experiments, such as identity-strengthening training based on narrative therapy, can be used to manipulate the independent variable and examine its dose-response effects on the mediator and outcome variables. These approaches would enhance the rigor of causal inference.

Ecological validity and cognitive bias in self-report data: Although Harman’s single-factor test was conducted to control for common method bias, self-report data remain subject to two major limitations: (1) Social desirability bias: Individuals with high social anxiety may underreport symptom severity due to stigma-related concerns (Podsakoff et al., 2003). (2) Cognitive processing bias: Anxiety-prone individuals often show attentional fixation on threat cues, such as a 50% increase in first fixation duration on negative facial expressions as revealed by eye-tracking studies, potentially distorting their subjective evaluation of social interactions (Mogg and Bradley, 2002). Future studies should adopt a multimodal assessment framework. (1) Behavioral level: Virtual reality-based social tasks can be used to quantify the frequency and intensity of avoidance behaviors. (2) Physiological level: Simultaneous monitoring of salivary cortisol and heart rate variability can capture real-time changes in the stress response. (3) Neural level: Functional MRI can be used to examine how individuals with high self-identity exhibit increased prefrontal inhibition of amygdala activation when processing negative social feedback (Goldin et al., 2012). Such multimodal data integration can overcome the limitations of self-report methods and enhance the ecological validity of findings.

Theoretical blind spots in individual heterogeneity and subtype-specific mechanisms: Existing models are largely based on a variable-centered paradigm that assumes that all individuals follow the same underlying mechanism. However, network analyses have suggested that the clinical symptoms of social anxiety are driven by distinct etiological networks (Fisher et al., 2018). Future research should adopt a person-centered approach, such as latent profile analysis or dynamic systems modeling, to identify meaningful subgroups. Mixed-method designs, such as combining ecological momentary assessments (EMA) to track daily anxiety fluctuations with retrospective interviews to reveal major causes, may be used to elucidate subtype-specific mechanisms. These findings may inform precision-targeted interventions such as exposure therapy for trauma-based subtypes or cognitive flexibility training for cognitively driven subtypes.

Theoretical omission of positive psychological resources and limitations in cultural generalizability: This study focused primarily on the amplifying effects of risk factors on anxiety, while overlooking protective constructs such as psychological resilience and self-compassion. Positive psychology suggests that individuals with high resilience can engage in meaning reconstruction, transforming social challenges into opportunities for personal growth rather than interpreting them as threats (Fredrickson, 2001). Future research should consider developing a dual-pathway integrative model to examine the relationship between risk and protective factors. It is important to test the cultural boundary conditions of these mechanisms. In collectivist cultures, family support may directly buffer anxiety by enhancing a sense of belonging. Self-efficacy and independent coping strategies may play a more prominent role in individualistic cultures (Rose and Kitayama, 1991). Cross-cultural comparisons may help delineate the generalizability and contextual applicability of intervention strategies.

While the structural equation model demonstrated good fit indices, it is important to consider the methodological trade-offs associated with item parceling. Item parceling was used in this study to enhance model parsimony and statistical power, particularly given the moderate sample size. Parcels were created based on theoretical coherence and internal consistency, following established recommendations in SEM literature. Cronbach’s alpha values for all scales exceeded 0.70, indicating acceptable reliability. However, we acknowledge that item parceling may mask problematic items and reduce the granularity of measurement, potentially inflating parameter estimates. To address this, we carefully examined individual item properties prior to parceling, and note that all indicators met psychometric criteria for internal consistency. Future studies with larger samples are encouraged to conduct item-level confirmatory factor analyses to validate the structure more precisely and to determine whether parceling remains necessary.

### 5.1 Key path for future research

First, EMA should be used to capture real-time fluctuations in self-identity and examine their predictive effects on daily anxiety. Second, physiological indicators such as cortisol levels should be integrated to quantify the biological buffering effects of social support. Third, network analysis methods may help reveal the distinct regulatory roles of different sources of social support (e.g., family, peers, and teachers) within the moderating mechanism. Notably, the widespread adoption of online social interaction due to the COVID-19 pandemic may have altered the functional dynamics of social support. Future research should examine the intervention potential of virtual support systems.

## 6 Conclusion

Self-identity negatively predicts social anxiety; FNE partially mediates the effect of self-identity on social anxiety; and the latter segment of the mediation pathway (from FNE to social anxiety) in the relationship between self-identity and social anxiety is moderated by perceived social support. These findings provide a theoretical foundation for developing an integrated intervention framework that combines cognitive restructuring (through self-identity and FNE) with environmental empowerment (through social support). Self-identity development should be incorporated into the main curriculum of mental health education. In addition, AI-driven systems for dynamic monitoring of social support should be developed to enable full-cycle prevention and early intervention of anxiety risk.

## Data Availability

The datasets presented in this study can be found in online repositories. The names of the repository/repositories and accession number(s) can be found in this article/supplementary material.
